# Evaluation of the relationship between the range of radiation-induced lung injury on CT images after IMRT for stage I lung cancer and dosimetric parameters

**DOI:** 10.1080/07853890.2020.1869297

**Published:** 2021-01-12

**Authors:** Tomohiro Itonaga, Shinji Sugahara, Ryuji Mikami, Tatsuhiko Saito, Takafumi Yamada, Masahiko Kurooka, Sachika Shiraishi, Mitsuru Okubo, Kazuhiro Saito

**Affiliations:** Department of Radiology, Tokyo Medical University Hospital, Shinjuku, Japan

**Keywords:** IMRT, RILI, DVH, NSCLC, DIR, stage I

## Abstract

**Background:**

This study evaluated the correlation between radiation-induced lung injury (RILI) and dosimetric parameters on computed tomography (CT) images of stage I non-small cell lung cancer (NSCLC) patients undergoing intensity-modulated radiotherapy (IMRT).

**Materials and methods:**

Sixty-three stage I NSLC patients who underwent IMRT were enrolled in the study. The patients underwent CT within 6 months (acute phase) and 1.5 years (late phase) after radiotherapy. These were fused with the planned irradiation CT. The range of RILI was measured from 10% to 100%, with an IC in 10% increments.

**Results:**

The median interval from completion of radiotherapy to acute and late phase CT was 92 and 440 days, respectively. The median RILI ranges of the acute and late phases were in the 80% (20–100%) and 70% dose regions (20–100%), respectively. The significantly narrower range of RILI when lung V20 in the acute phase was less than 19.2% and that of V5 in the late phase was less than 27.6% at the time of treatment planning.

**Conclusions:**

This study showed that RILI occurred in a localized range in stage I NSCLC patients who underwent IMRT. The range of RILI was correlated with V20 in the acute phase and V5 in the late phase.

**KEY MESSAGES**
- RILI correlated with V20 in acute and V5 in late phase.
- The shadow of RILI occurred in 80% dose region in acute and 70% in late phase.
- No relationship exists between radiographic changes in RILI and PTV volume.

## Introduction

Radiation-induced lung injury (RILI) is one of the most frequent toxicities in patients with stage I non-small cell lung cancer (NSCLC) treated with radiotherapy [1,2]. RILI acutely presents as radiation pneumonia (RP) within six months of completion of radiotherapy. In the chronic phase, it develops as radiation pulmonary fibrosis (RF) [3,4]. This phenomenon is caused by a combination of direct radiation cytotoxicity to the normal lung tissue and the complex mechanisms of radiation-induced cells, mediators and signalling pathways [5,6]. The estimated incidence of RILI varies between studies because it depends on the selected endpoint, clinical stage, lung dose and radiotherapy technique. Among patients receiving radiotherapy for lung cancer, approximately 5–30% had clinical symptoms while almost all had radiologic findings [7–9].

Two decades ago, locally advanced lung cancer was treated with three-dimensional conformal external beam radiation therapy (3D-CRT). 3D-CRT has a dose homogeneity within the irradiated field, and the imaging features of RP have relatively sharp edges that coincide with the beam angle rather than the anatomical boundaries. Advances in radiotherapy techniques have resulted in intensity-modulated radiation therapy (IMRT) for lung cancer, which precisely irradiates complex geometric targets and reduces the dose to adjacent organs at risk (OARs). IMRT differs from 3D-CRT in that it is characterized by dose heterogeneity within the irradiation field. For this reason, it is known that the morphology of RILI after lung cancer using IMRT differs from that after 3D-CRT [10]. However, there are few papers summarizing the range and morphological changes of RILI after the use of IMRT, and it is important to differentiate RILI from pneumonia or local recurrence. We studied the correlation between RILI and lung dose in patients with stage I NSCLC treated with IMRT using the same protocol. If the morphology and extent of RILI can be predicted at the time of radiation treatment planning, this could provide useful information for post-treatment imaging.

## Materials and methods

### Patient eligibility

This study was performed as a sub-analysis of a previous study and was approved by the ethical committee of Tokyo Medical University Hospital in Tokyo, Japan (IRB number SH4121). The patient's eligibility criteria were as previously reported [11]. From March 2011 to March 2018, 92 patients, who were diagnosed with 94 stage I NSCLC tumours according to the seventh edition of the UICC TNM classification, were enrolled and underwent IMRT. The eligibility criteria in this study excluded those with fulfilling the following conditions: (i) patient follow-up < 12 months, (ii) patients with local recurrence or pleural effusion within 12 months and (iii) tumours with a target registration error (TRE) of 3 mm or more when evaluating the accuracy of image fusion. Sixty-three patients, totalling 65 tumours, were enrolled in the study. Of these patients, 39 were male and 24 were female, and the median age was 79 years old (range, 56–93 years).

### Radiotherapy methods

The details of the treatment procedure were previously described in the phase II study [12]. All patients were immobilized in the supine position using a body immobilization shell system (Pelvicast; Orfit Industries n.v., Wijnegem, Belgium). Images of the radiotherapy planning were taken using a 16 multislice computed tomography (CT) (Aquilion LB, Toshiba Medical Systems, Tokyo, Japan). The prescribed dose was set at 75 Gy in 30 fractions covering 95% of the planning target volume. The IMRT plan was created using a treatment planning system (Xio ver. 4.6 system, Elekta AB, Stockholm, Sweden). Usually, five non-coplanar beams, three coplanar beams from the caudal direction, and two non-coplanar beams from the cranial direction, were arranged to reduce the OARs, such as the lung, spinal cord and the oesophagus. Chemotherapy was not performed during radiotherapy.

### Follow-up

Patients were followed up every three months after the completion of radiation treatment. They received medical consultation and underwent chest CT scans every three months within two years and then every six months thereafter. Lung imaging was performed using a LightSpeed VCT 64-slice CT scanner (GE Healthcare, Waukesha, WI) at a slice thickness of 5 mm. The parameters for CT acquisition were as follows: helical pitch, 1.375:1; beam collimation, 20 mm; and reconstruction, 5 mm. When recurrence was suspected, an immediate follow-up CT or PET-CT was performed. The final judgement regarding recurrence was made during the cancer board held every week. The severity of RILI was assessed according to the Common Terminology Criteria for Adverse Events (CTCAE), ver.4.0.

### Measurement method

RILI was evaluated via CT imaging after radiotherapy. The acute and late phases of RILI were evaluated from the CT imaging results at one to six months and one to 1.5 years after completion of radiotherapy, respectively. The CT images of the lungs in the acute and late phases were transferred to MIM Maestro (MIM Maestro ver. 6.1, MIM Software Inc., Cleveland, OH). We set the target imaging as treatment plan, souse imaging as follow-up CT, and performed deformable image registration (DIR) limited to the irradiation field. The DIR algorithm in MIM Maestro uses an intensity-based free-form DIR. To evaluate the accuracy of the DIR, five TRE were set along the pulmonary vessels near the irradiation field in both target imaging and souse imaging. Previous studies have shown that the spatial accuracy of DIR was higher when the TRE was less than 3 mm. Therefore, in the present study, cases with a tumour greater than 3 mm were excluded [13,14]. This reduced the deviation caused by the assessor. Treatment planning and follow-up CT were overlaid using the MIM Maestro, and the range of RILI was measured from 10% to 100% with an isodose curve (IC) in increments of 10%. In the absence of RILI on CT, the patient was evaluated as 100%. The dosimetric parameters of the lung were measured from a dose–volume histogram (DVH). Normal lung volume was measured using gross tumour volume exclusion from the ipsilateral and bilateral lungs. From DVH of the lungs, V5, V10, V20 and V30 were measured.

The morphological evaluation of RILI on CT appearance was judged according to Linda’s classification [15]. Acute phase CT was classified into the following five patterns: (1) diffuse consolidation, (2) diffuse ground-glass opacity (GGO), (3) patchy consolidation and GGO, (4) patchy GGO and (5) no change. The late phase CT was classified into the following four patterns: (1) modified conventional pattern, (2) mass-like pattern, (3) scar-like pattern and (4) no changes. The appearance pattern classification from the CT data was performed by two radiologists blinded to the clinical data. Analysis of the range and morphological evaluation of RILI on CT was performed by two radiologists (T.Y. and T.S., with 10 and 8 years of experience, respectively) blinded to the clinical data.

### Statistical analyses

The Spearman correlation was used to evaluate the correlation between the RILI range on CT and dosimetric factors in the lungs. Continuous data were compared using the Mann–Whitney *U* test. The optimal cut-off value for analysing the relationship between the range of RILI on CT images and dosimetric parameters was determined using the receiver operating characteristics (ROC) curve. After dividing the two groups using cut-off values, the range of RILI was compared using Fisher's exact test. All statistical analyses were two-tailed, and a *p* value of <.05 represented statistical significance. R software (v. 3.1.0, R Foundation for Statistical Computing, Vienna, Austria) was used for all statistical calculations.

## Results

Patient and tumour characteristics are summarized in Table 1. The median tumour diameter was 21 mm (range: 10–45 mm), and the PTV volume was 20.74 cm^3^ (range: 5.21–103.49 cm^3^). The median interval from completion of radiotherapy to acute and late phase CT was 92 days (range: 22–183 days) and 440 days (range: 364–599 days), respectively. The mean dosimetric values of V5, V10, V20 and MLD are shown in Supplemental Material 1. Two patients experienced grade 2 radiation pneumonitis according to the CTCAE version 4.0, and none of them experienced toxicities of grade 3 or greater.

**Table 1.** Patient and tumour characteristics.

Factors		No.
Sex	Male	39
	Female	24
Age	Median	79
	Range	56–93
Location	Right	25
	Left	40
Location	Upper	41
	Middle	3
	Lower	21
Pathology	Adenocarcinoma	18
	Squamous cell carcinoma	9
	NSCLC	5
	Pathology unproven	33
Maximum tumour diameter	Median	21
	Range	10–45
Solid tumour component diameter	Median	16
	Range	0–41
T factor of TNM 8th	Tis	3
	T1a	13
	T1b	27
	T1c	12
	T2a	9
	T2b	1

The morphological evaluation of RILI on CT appearance and time course are shown in Table 2. The most common changes in the patterns of CT imaging from acute to late phases were “patchy consolidation and GGO” to “modified conventional pattern” (14 cases), “diffuse consolidation” to “modified conventional pattern” (11 cases) and "No change" to "No change" (10 cases).

**Table 2.** Morphological evaluation of RILI on CT appearance.

Acute		Late	
CT appearance	Total no. (%)	CT appearance	Total no.
Diffuse consolidation	18 (27.7%)	Modified conventional pattern	37 (56.9%)
Diffuse GGO	8 (12.3%)	Mass-like pattern	6 (9.2%)
Patchy consolidation and GGO	20 (30.8%)	Scar-like pattern	12 (18.5%)
Patchy GGO	1 (1.5%)	No changes	10 (15.4%)
No changes	18 (27.7%)		

**Figure 1.**
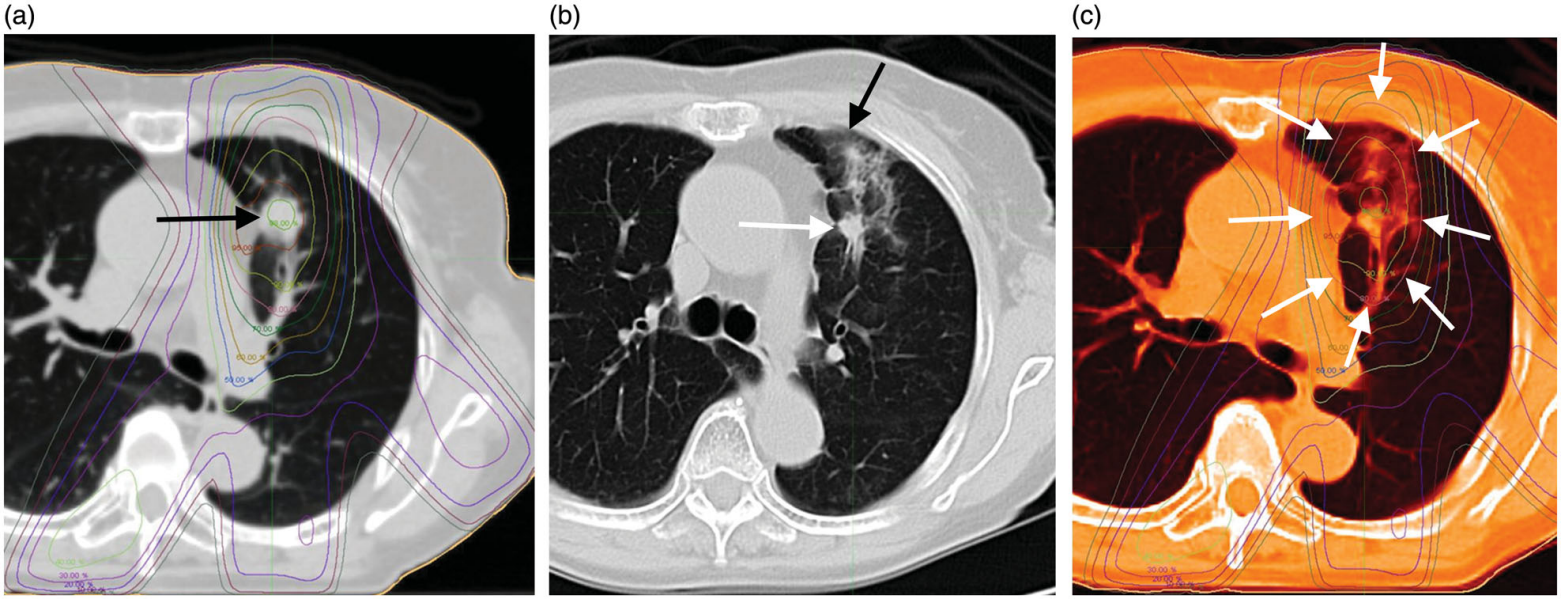
(a) The case is an 85-year-old woman with a 24-mm-diameter squamous cell carcinoma of the left upper lobe S3 lung (black arrow). (b) This shadow was diagnosed as a "Patchy consolidation and ground glass opacity (GGO)" in acute phase CT imaging at 114 days after radiotherapy (black arrow: ground glass opacity, white arrow: consolidation). (c) Treatment planning and acute computed tomography (CT) imaging were fused with MIM Maestro. The shadow of RILI was found in the 80% dose region (white arrows).

**Figure 2.**
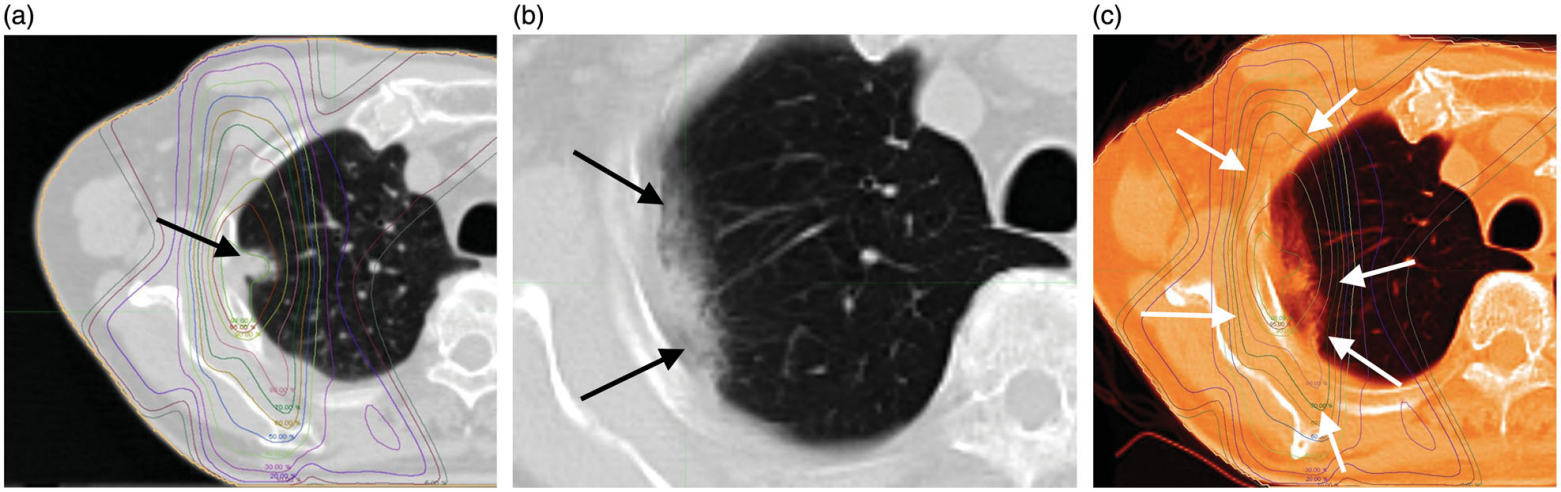
(a) An 84-year-old man with a 14-mm-diameter adenocarcinoma in the right upper lobe S2 of the lung (black arrow). (b) This shadow appeared as a "Scar-like pattern" in late phase computed tomography (CT) imaging at 559 days after radiotherapy (black arrows). (c) Treatment planning and acute CT imaging were fused with MIM Maestro. The shadow of RILI was found in the 70% dose region (white arrows).

There was no evidence of RILI appearing outside the irradiation field or in the contralateral lung. The median range of the acute and late phases were in the 80% (range: 20–100%, Figure 1) and 70% (range: 20–100%, Figure 2) dose regions, respectively. There were no significant differences in the extent of acute and late pneumonia based on age (>75 years) or sex. The Spearman correlation coefficients between the RILI of acute and late phase CT and dosimetric parameters are listed in Supplemental Material 2. The extent of RILI in the acute and late phases was negatively correlated with the dose parameters of the bilateral and ipsilateral lung.

The Mann–Whitney *U* test was performed to determine the relationship between lung dosimetric parameters and cases without RILI on acute and late CT images, respectively (Supplemental Material 3). The lung dose parameters were significantly lower in cases with "No change" in both the acute and late phases. Cases with consolidation on acute CT imaging had a significantly wider range of RILI in both acute (*p*<.001) and late (*p*=.01) phases. The optimal cut-off value for the range of RILI in patients with "no change" in the acute phase was correlated with 90% dose in the late phase (range: 40–100%, AUC: 0.81), when analysed using ROC curve. The optimal cut-off values for the dose parameters were analysed by dividing the two groups using the respective cut-off values in the acute and late phases (Supplemental Material 4). We excluded bilateral lung data from the analysis because the beam was positioned such that the exiting beam did not enter the contralateral lung. We also excluded it from the analysis because we used a compensator-based IMRT rather than VMAT, which recorded lower MLD values (median: ipsilateral = 1.4 Gy, bilateral = 0.3 Gy). Lung V20 in the acute phase and V5 in the late phase were significantly associated with the range of RILI. The range of RILI in the acute phase was 80% in cases with V20 < 19.2%, and it was 60% in the rest of the cases. In the late phase, the rate was 80% in cases with V5 < 27.6% and 60% in the rest of the cases. The PTV volume was not associated with the range of RILI in either the acute or late phase.

## Discussion

We evaluated the range of RILI on two CT scans of stage 1 NSCLC within six months (acute phase) and 1.5 years (late phase) after radical radiotherapy with IMRT. This is a single-centre study using the same treatment policy, dose formulation and dose constraints. It is an optimal condition for measuring the range of RILI.

In our study, RILI was consistent with 80% and 70% of the ICs in acute and late phase CT, respectively. There are few studies on the extent of RILI after radical radiotherapy for lung tumours. Previous studies have reported that with radical radiotherapy using 3D-CRT at 2 Gy/fraction, RILI commonly caused minor radiological changes at <20 Gy. A gradual increase in the 30–50 Gy range was noted, and there were greater changes observed at 50 Gy and above [16,17]. Aoki et al. reported that RILI appeared in a median range of 24 Gy in a study of hypofractionated SBRT (12 Gy/fraction) for solitary lung tumours [9]. Knoll et al. reported that 95% of RILI occurred within the 15 Gy IC and 90% occurred within the 20 Gy IC surrounding the RILI for patients with lung cancer who underwent hypofractionated SBRT (9–18 Gy per fraction) [18]. Converting the percentage of the IC in our study to Gy revealed that RILI occurred at the 60 Gy range in the acute phase and at 52.5 Gy in the late phase. We calculated the biological equivalent dose and equivalent dose in 2 Gy fraction (EQD2) based on *α*/*β* = 3 to compare the range of RILI with previous studies (Supplemental Material 5) [19]. Although the comparison does not consider the difference in PTV volume, our study showed that RILI appeared in the relatively high dose range. RILI was correlated with higher radiation doses. Creating dose distribution with high dose concentration is one advantage of IMRT [15].

A limitation of previous studies was that the extent of RILI was evaluated via naked eye assessment and lacked an objective assessment method. We considered using DIR to address this problem. DIR is a matching process that generates vectors that move the position of each pixel in the source imaging to the position of the corresponding pixel in the target image and deforms the image to match the target image. The American Association of Physicists in Medicine reported a task group report 132 on DIR, and the clinical use of DIR has been rapidly spreading [20]. It is important to ensure the accuracy of DIR, and we adopted TRE, a quantitative evaluation using anatomical indices. TRE is a method in which index points are set at positions that show the same anatomical features in source imaging and target imaging. The coordinate difference between the two points is evaluated as the error of DIR [14]. If the points of both the source imaging and the target imaging completely match, the TRE becomes zero. Previous studies have shown that the spatial accuracy of DIR was high when TRE was less than 3 mm, and this criterion was adopted in the present study [21–23]. Therefore, it is unlikely that we are seeing an effect of reduced lung volume. Although, RILI was present in 80% of ICs in the acute phase and 70% in the late phase. To the best of our knowledge, this is the first study using DIR to measure the range of RILI.

The morphological changes on the CT imaging of RILI during the acute and late phases were compared with other studies (Table 3). Previous studies have employed hypofractionated radiotherapy that increases the dose per fraction in order to achieve a higher treatment effect with fewer treatments. Instead, our study adopts 2.5 Gy per fraction, which is close to the conventional dose (2 Gy per fraction); however, there was no significant difference in the morphology of RILI compared to previous studies. This finding suggests that differences in dose per fraction may not contribute to the morphological differences in RILI. The incidence of clinically observed CTCAE grade 2 or higher pneumonia was 3% in the study, but 72% and 85% had radiological findings on CT imaging of acute and late phases, respectively. The incidence of severe pneumonia in this study was lower than the average incidence reported in the literature. This is a problem in studies involving cases without local recurrence for at least one year [28]. Previous studies have reported that changes in RILI on acute phase CT images predict the severity of the late phase CT [25,27]. The evaluation of RILI on CT imaging in previous studies was limited because the definition of "severe" was assessed subjectively by the observer. In our study, we reported that changes in RILI with consolidation on acute phase CT images predicted an increase in the range of RILI on late phase CT.

**Table 3.** Summary of studies reporting radiological changes after radiotherapy for lung cancers.

	Prescribed dose (lesion numbers)	Acute					Late			
		Patchy consolidation/ GGO	Patchy GGO	Diffuse GGO	Diffuse consolidation	No change	Modified conventional pattern	Mass-like patterns	Scar-like pattern	No change
Trovo et al. [24]	54–60 Gy/3 Fr (59)	33%	6%	21%	27%	21%	44–54%	20–28%	14–16%	12–20%
	45–48 Gy/3–6 Fr (11)									
Dahele et al. [25]	60 Gy/3 Fr (24)	24%	6%	7%	16%	46%	71%	7%	11%	11%
	60 Gy/5 Fr (26)									
	60 Gy/8 Fr (11)									
Yamamoto et al. [26]	40 Gy/4 Fr (37)	31–38%	0%	0%	18–33%	22–31%	49–57%	16–22%	24%	2–4%
	48 Gy/4 Fr (12)									
Kimura et al. [27]	54 Gy/14 Fr (6)	15%	2%	12%	39%	33%	62%	17%	21%	0%
	60 Gy/10 Fr (8)									
	60 Gy/8 Fr (4)									
	50 Gy/5 Fr (6)									
	48 Gy/4 Fr (18)									
Our study	75 Gy/30 Fr (63)	31%	2%	12%	28%	28%	57%	9%	19%	15%

Although many dosimetric parameters of the lung have been reported as prognostic factors in studies investigating RILI after SBRT, many studies have reported that V20 is commonly correlated with the severity of RILI [29–31]. Our study showed that V20 was the most correlated variable for RILI in the acute phase and V5 was the most correlated variable for RILI in the late phase. A previous study reported that V5Gy, V20Gy and mean lung dose were significantly correlated with severe RILI in cases with consolidation on acute CT images [19]. Our study showed a significantly narrower range of RILI when V5 was less than 27.6% and V20 was less than 19.16% at the time of treatment planning. A meta-analysis of the relationship between dosimetric parameters of SBRT and risk of RP in lung tumours showed that MLD ≥ 4.7 Gy, V5 ≥ 26.8%, V10 > 12% and V20 ≥ 5.8% were associated with RP risk [32]. This correlates with the results of our study and may be an indicator for the prevention of severe RILI. Similar to previous studies, we found no relationship between radiographic changes in RILI and PTV volume [24–26]. Matsuo et al. reported that RILI after SBRT for lung cancer (median PTV volume = 32 cm^3^) was significantly higher in the group with a PTV volume greater than 37.7 cm^3^ [29]. The median amount of PTV in our study was as low as 20.74 cm^3^, and the group with more than 37.7 cm^3^ was as low as 26%. In stage I lung cancer with small amount of PTV, the range of RILI on CT appearance may correlate with the lung dosimetric parameters.

Our study has several limitations. First, this study is the result of IMRT using 2.5 Gy per fraction. For patients with inoperable stage I NSCLC, hypofractionated SBRT with >10 Gy per fraction is the mainstream treatment option. Thus, it is necessary to verify whether the results of this study are comparable with those of hypofractionated SBRT. Second, this study was retrospective, and the sample size was limited. Third, our study included patients who underwent IMRT for stage 1 NSCLC, and there were few cases with CTCAE grade 2 or higher. Therefore, the relationship between symptomatic RILI and the range of RILI on CT is unclear.

## Conclusions

In conclusion, this study showed that RILI occurred in a localized range in patients with stage I NSCLC who underwent IMRT. It suggested that an increase in lung dose parameters was correlated with an increase in the range of RILI on CT images. The range of RILI was correlated with V20 in the acute phase and V5 in the late phase. Lung V5 of less than 27.6% and a V20 of less than 19.2% at the time of treatment planning showed a narrower range of RILI in the acute and late phases.

## Supplementary Material

Supplemental Material

## Data Availability

The authors confirm that the data supporting the findings of this study are available within the article and its supplementary materials.
