# Supplementary material for: Evaluation of the relationship between the range of radiation-induced lung injury on CT images after IMRT for stage I lung cancer and dosimetric parameters
Source: Ann Med. 2021 Jan 12;53(1):267–73. doi: 10.1080/07853890.2020.1869297 (PMC7877951; doi:10.1080/07853890.2020.1869297)
Supplement: Supplemental Material [file IANN_A_1869297_SM0080.docx]

Supplementary Material 1. Median and range for the dose-volume histogram metrics

| **Factor** | Ipsilateral  Median difference | Bilateral  Median difference |
| --- | --- | --- |
| V5 (%) | 33.20  (10.17- 77.31) | 18.8  (4.83-41.82) |
| V10 (%) | 27.69  (7.80- 60.09) | 13.4  (4.04-29.28) |
| V20 (%) | 16.50  (4.34- 39.21) | 8.11  (2.22-18.37) |
| V30 (%) | 9.95  (2.25- 30.51) | 5.03  (1.15-14.1) |
| MLD (Gy) | 1.41  (0.08- 12.93) | 0.25  (0.05-2.79) |

Supplementary Material 2. The Spearman correlation coefficients between the RILI of acute and late phase CT and dosimetric parameters

| **Factor** | **Ipsilateral** | | **Bilateral** | |
| --- | --- | --- | --- | --- |
|  | Acute RILI | Late RILI | Acute RILI | Late RILI |
| **V5 (%)** | Srcc = -0.31  P= 0.01 | Srcc = -0.44  P<0.01 | Srcc = -0.29  P= 0.02 | Srcc= -0.45  P<0.01 |
| **V10 (%)** | Srcc = -0.31  P= 0.01 | Srcc = -0.40  P<0.01 | Srcc= -0.29  P= 0.02 | Srcc= -0.45  P<0.01 |
| **V20 (%)** | Srcc = -0.30  P= 0.02 | Srcc = -0.34  P= 0.01 | Srcc= -0.32  P= 0.01 | Srcc= -0.42  P<0.01 |
| **V30 (%)** | Srcc = -0.24  P= 0.05 | Srcc = -0.26  P= 0.04 | Srcc= -0.24  P= 0.06 | Srcc= -0.34  P<0.01 |
| **MLD (Gy)** | Srcc = -0.30  P= 0.01 | Srcc = -0.46  P<0.01 | Srcc= -0.28  P= 0.02 | Srcc= -0.39  P<0.01 |

*Spearman's rank correlation coefficient = Srcc

Supplementary Material 3. The relationship between lung dosimetric parameters and cases without RILI on acute and late CT images by the Mann–Whitney U test

| Factor | Ipsilateral | | Bilateral | |
| --- | --- | --- | --- | --- |
|  | Acute  “RILI” VS “No RILI” | Late  “RILI” VS “No RILI” | Acute  “RILI” VS “No RILI” | Late  “RILI” VS “No RILI” |
| V5 (%) | M:39.7% VS 26.4%  P= 0.04 | M:36.4% VS 18.6%  P<0.01 | M: 20.5% VS 14.2%  P= 0.03 | M: 20.5% VS 10.9%  P<0.01 |
| V10 (%) | M:29.1% VS 20.8%  P= 0.04 | M:28.8% VS 14.4%  P<0.01 | M: 13.9% VS 10.9%  P= 0.05 | M: 14.4% VS 7.8%  P<0.01 |
| V20 (%) | M:18.3% VS 13.2%  P= 0.02 | M:17.6% VS 9.1%  P<0.01 | M: 9.3% VS 7.2%  P= 0.02 | M: 9.1% VS 5.5%  P=0.01 |
| V30 (%) | M:10.8% VS 8.4%  P= 0.02 | M:10.8% VS 6.5%  P= 0.01 | M: 5.8% VS 4.6%  P= 0.03 | M: 5.2% VS 3.9%  P= 0.02 |
| MLD (Gy) | M:1.8% VS 0.4%  P= 0.04 | M:1.7% VS 0.3%  P<0.01 | M: 0.3% VS 0.1%  P= 0.02 | M: 0.3% VS 0.1%  P= 0.01 |

M: Median value

Supplementary Material 4. Optimal cut-off values and p value of “no change” RILI

1. Acute phase

|  | AUC | Cut-off value | P-value |
| --- | --- | --- | --- |
|  |  |  |  |
| iV5(%) | 0.63 | 39.58 | P=0.02 * |
| iV10(%) | 0.63 | 28.19 | P=0.04 * |
| iV20(%) | 0.63 | 19.16 | P=0.01 * |
| iV30(%) | 0.61 | 10.46 | P=0.04 * |
| PTV volume(cc) | 0.48 | 18.28 | P=0.80 |

1. Delay phase

|  | AUC | Cut-off value | P-value |
| --- | --- | --- | --- |
|  |  |  |  |
| iV5(%) | 0.73 | 27.60 | P<0.01 * |
| iV10(%) | 0.70 | 21.74 | P=0.01 * |
| iV20(%) | 0.67 | 15.20 | P=0.05 |
| iV30(%) | 0.61 | 10.46 | P=0.09 |
| PTV volume(cc) | 0.60 | 30.30 | P=0.07 |

Asterisks (*): statistical significance, i: ipsilateral, PTV: planning target volume

Supplementary Material 5. Comparison of our study with previous studies on the range of RILI

|  | | Median f/u | Radiation technique | Treatment  dose | Median dose of RILI occurrence | BED | EQD2 |
| --- | --- | --- | --- | --- | --- | --- | --- |
| Our study | Acute | 3 M | IMRT | 75 Gy/ 30Fr | 60 Gy | 100 Gy | 60 Gy |
|  | Delay | 14.7 M |  |  | 52.5 Gy | 83.13 Gy | 49.88 Gy |
| Aoki | | 4 M | SBRT | 48Gy/ 4Fr | 24 Gy | 72 Gy | 43.2 Gy |

* Knoll et al. report dose per fraction differently and cannot be compared.
